# Epidemiological Characteristics and Mental Health Disparities Between War-Displaced Ukrainian and Host-Country People Living with HIV in Slovakia: A Cross-Sectional Study

**DOI:** 10.3390/healthcare14081093

**Published:** 2026-04-20

**Authors:** Kristína Doležalová, Ricardo Massmann, Ľubomír Soják, Lucia Kročková, Matej Bendžala, Eliška Marešová, Peter Mihalov, Soňa Kašická, Mária Borsányiová, Jakub Vallo, Peter Sabaka

**Affiliations:** 1 National Reference Centre for HIV/AIDS Prevention, Slovak Medical University, 833 03 Bratislava, Slovakia; kristina.dolezalova@szu.sk (K.D.); maria.borsanyiova@szu.sk (M.B.); 2 Department of Infectology and Geographical Medicine, Faculty of Medicine, Comenius University in Bratislava, 811 08 Bratislava, Slovakia; medicinemassmann@gmail.com (R.M.); lubomir.sojak@kr.unb.sk (Ľ.S.); luciakrockova.kigm@gmail.com (L.K.); mbendzala@gmail.com (M.B.); elikrajcovicova@gmail.com (E.M.); mihalovpeter@gmail.com (P.M.); 3 Department of Labour Law and Social Security Law, Faculty of Law, Comenius University in Bratislava, 810 00 Bratislava, Slovakia; sona.kasicka@flaw.uniba.sk; 4 Emergency Department, University Hospital Bratislava, 811 07 Bratislava, Slovakia; jakub.vallo@gmail.com

**Keywords:** HIV, PLHIV, mental health, war-displaced persons, Ukraine refugee crisis, gender-specific outcomes

## Abstract

**Highlights:**

**What are the main findings?**

- War-displaced Ukrainian people living with HIV achieved high rates of viral suppression and combined antiretroviral therapy coverage, comparable to host-country patients within the Slovak healthcare system.
- While overall mental health scores did not differ between cohorts, a significant gender-based disparity was identified within the Ukrainian group, where women exhibited higher depressive symptom severity than men.

**What are the implications of the main findings?**

- The study demonstrates that structured European healthcare systems can effectively maintain biomedical stability for HIV-positive refugees even during active conflict and displacement.
- Clinical care for displaced populations must move beyond a purely biomedical approach to integrate longitudinal, gender-sensitive mental health screening that addresses the specific psychosocial vulnerabilities of displaced women.

**Abstract:**

**Background**: The full-scale Russian invasion of Ukraine in 2022 triggered the largest displacement crisis in Europe in recent decades. Displacement may affect both clinical outcomes and mental health among people living with HIV (PLHIV). Evidence comparing displaced PLHIV with host-country patients within the same healthcare system remains limited. This study aimed to compare epidemiological characteristics, clinical staging, and mental health outcomes between war-displaced Ukrainian PLHIV and Slovak PLHIV receiving care in the same clinical setting, with particular attention to sex-specific differences. **Methods**: This cross-sectional study included 137 PLHIV receiving care at the HIV/AIDS Centre, University Hospital Bratislava, Slovakia (69 from Ukraine and 68 from Slovakia). Anxiety and depressive symptoms were assessed using the Generalized Anxiety Disorder-7 (GAD-7) and Patient Health Questionnaire-9 (PHQ-9) scales. Scores were categorized into three severity groups (0–4, 5–9, ≥10). **Results**: Age distribution was comparable between cohorts (*p* = 0.2438). Transmission patterns differed substantially: heterosexual transmission predominated among Ukrainian participants, whereas men who have sex with men (MSM) transmission predominated among Slovak men (*p* < 0.001). Ukrainian patients were more frequently classified in CDC stage C, while Slovak patients more often presented in stage A. The combined antiretroviral therapy coverage was 100% in both cohorts and viral suppression rates were high (HIV RNA < 200 copies/mL: 91.3% in Ukraine vs. 94.1% in Slovakia). Overall anxiety and depressive symptom severity did not differ significantly between cohorts (GAD-7 *p* = 0.4145; PHQ-9 *p* = 0.7661). However, within the Ukrainian cohort, women demonstrated higher depressive symptom severity compared with men (*p* = 0.0478). **Conclusions**: War-displaced Ukrainian PLHIV achieved comparable biomedical outcomes to host-country patients within a structured healthcare system. However, depressive vulnerability emerged at the intersection of gender and displacement. These findings highlight the importance of integrating gender-sensitive mental health screening and psychosocial support into routine HIV care for displaced populations.

## 1. Introduction

Human immunodeficiency virus (HIV) infection remains a major global public health challenge despite substantial progress in combined antiretroviral therapy (cART) and reductions in AIDS-related mortality. In 2024, approximately 40.8 million people were living with HIV worldwide (37.0–45.6 million) [1]. Beyond virological control, mental health has emerged as a critical determinant of long-term outcomes among people living with HIV (PLHIV). Depression and anxiety are highly prevalent and consistently associated with poorer adherence, reduced virological suppression, and increased mortality [2,3,4,5]. The full-scale Russian invasion of Ukraine in February 2022 triggered the largest humanitarian crisis within Europe in the 21st century [6,7]. Ukraine has one of the highest HIV prevalence rates in Eastern Europe, with a historically concentrated epidemic and regional disparities in access to care [8,9]. Forced migration and armed conflict introduce additional structural determinants that may amplify both clinical and psychosocial vulnerability. Displacement disrupts housing, employment, social networks, and access to healthcare, creating chronic stress exposures beyond acute trauma [10,11]. To systematically evaluate these dynamics, this study is grounded in two complementary frameworks. First, structural vulnerability theory emphasizes that political violence and institutional arrangements shape health outcomes beyond individual agency [12]. In humanitarian settings, the sudden disruption of healthcare infrastructure and the necessity of cross-border migration represent profound structural shocks [13]. Consequently, HIV-related stigma may intersect with displacement-related marginalization, producing layered health risks. While host-country healthcare systems may stabilize biomedical outcomes, displaced populations remain structurally vulnerable to ongoing psychosocial stressors. Second, intersectionality theory posits that overlapping identities—such as gender, migration status, and chronic illness—interact to produce compounded vulnerability [14]. Specifically, the concept of the ‘matrix of domination’ (originating from Patricia Hill Collins) serves as a critical framework for understanding how these interlocking systems of marginalization shape HIV-related outcomes in displaced populations today. Complementary to this is her notion of the ‘outsider within,’ which addresses individuals who are institutionally integrated—such as through a healthcare system—yet remain socially and culturally marginalized [15,16]. This dual lens is particularly relevant to the Ukrainian crisis, where women face unique structural pressures at the intersection of war-related trauma and HIV infection. Depression is globally more prevalent among women, including women living with HIV [2,3,17,18]. Women affected by armed conflict frequently experience magnified daily stressors, including caregiving strain, family separation, economic instability, and increased exposure to gender-based violence [11,14,19]. In conflict-affected populations, these daily stressors may mediate mental health outcomes more strongly than direct trauma exposure [16,20,21]. When the chronic management of HIV intersects with the trauma of displacement and gendered societal roles, the resulting mental health burden may diverge significantly from that of the host population. In clinical practice, the use of validated and standardized diagnostic instruments is essential for the objective quantification of psychological symptom severity. These brief, patient-reported outcome measures are designed to maximize diagnostic sensitivity while minimizing the administrative burden on healthcare providers. Their integration into the routine monitoring of patients with chronic conditions facilitates the early detection of affective disorders that might otherwise remain unrecognized [22,23]. Slovakia represents a highly relevant setting for this research. As a direct neighbor to Ukraine, it became a primary transit and destination country for displaced populations following the 2022 invasion [6,7]. Within the Slovak healthcare system, HIV care is highly centralized and managed through specialized regional centers, which ensure standardized, free-of-charge access to cART and comprehensive clinical monitoring [9]. Following the outbreak of the war, national emergency legislation and the activation of the EU Temporary Protection Directive granted temporary protection status to displaced Ukrainians, securing their uninterrupted access to specialized HIV care comparable to that of Slovak citizens [24]. Despite these rapid public health responses and the recognition of structural and intersectional frameworks, there is a notable gap in the European literature regarding the psychosocial integration of these patients. While recent studies have evaluated the clinical cascade and biomedical outcomes of Ukrainian migrants living with HIV—demonstrating high rates of viral suppression in host countries [13]—empirical comparisons focusing on mental health outcomes between HIV-positive refugees and host-country populations within a shared clinical setting remain exceptionally scarce. By explicitly anchoring our approach in these theories, we hypothesize that structural vulnerabilities manifest differently across gender and displacement status.

Beyond these frameworks, the interplay between HIV infection, mental health disorders, and social marginalization is increasingly conceptualized through the lens of syndemics. A syndemic framework moves beyond simple comorbidity by examining how large-scale social forces—such as armed conflict and forced displacement—interact with biological conditions to exacerbate health disparities [4]. In the context of the Ukrainian crisis, the ‘syndemic clustering’ of HIV, displacement-related trauma, and gender-based stressors may create a self-reinforcing cycle of vulnerability that challenges routine clinical management. Therefore, this study aimed to evaluate these complex interactions by comparing epidemiological characteristics, clinical staging, and mental health burden between war-displaced Ukrainian PLHIV and Slovak PLHIV treated in the same centralized setting. Specifically, we sought to determine if the high standard of biomedical care in Slovakia is mirrored by psychosocial stability, or if specific vulnerabilities—particularly sex-specific depressive patterns—emerge at the intersection of war-displacement and chronic illness.

## 2. Materials and Methods

### 2.1. Study Design and Participants

This cross-sectional study was conducted between 1 January 2024 and 31 January 2026 at the HIV/AIDS Centre, Department of Infectology and Geographical Medicine, University Hospital Bratislava, Slovakia. As the country’s largest tertiary HIV outpatient clinic, this facility serves as the primary national referral center, managing the vast majority of the Slovak HIV cohort. In the Slovak healthcare system, all HIV-related medical care—including long-term clinical monitoring (dispensary care) and cART is fully covered by mandatory public health insurance and provided to patients free of charge [9]. The Centre provides comprehensive, multidisciplinary HIV care, which includes routine virological monitoring, screening and treatment of coinfections (such as viral hepatitis and sexually transmitted infections), and the management of opportunistic diseases. Following the escalation of the conflict in Ukraine in 2022, the Centre assumed a pivotal role in the public health response, ensuring immediate and uninterrupted linkage to care for displaced PLHIV. Under the national temporary protection framework, these individuals were granted equivalent access, ensuring that all clinical and laboratory services, as well as cART, are provided to them free of charge.

Consecutive sampling of patients was applied. All patients attending routine follow-up visits during the study period were invited to participate. As of 31 January 2026, 860 people living with HIV were under follow-up at the centre (746 men and 114 women). The Slovak study sample closely reflected this sex distribution (59 men [86.76%] and 9 women [13.24%]). The Ukrainian study sample consisted of displaced persons with temporary residence in Slovakia who were receiving HIV care at the study site. Of the 69 Ukrainian displaced persons included in the study, 58 (84.06%) had been previously diagnosed and treated in Ukraine, whereas 11 (15.94%) were newly diagnosed in Slovakia. The sex distribution was broadly consistent with the national HIV profile in Ukraine [25], with 35 men (50.72%) and 34 women (49.28%) included in the sample. The study was conducted in accordance with the Declaration of Helsinki and approved by the Ethics Committee of the University Hospital Bratislava (protocol code 28/2023). Informed consent was obtained from all subjects involved in the study prior to participation.

### 2.2. Measures

Psychological symptom severity was assessed using two widely validated self-report instruments. To ensure accessibility and linguistic accuracy, validated Slovak and Ukrainian language versions of both questionnaires were utilized.

Generalized Anxiety Disorder-7 (GAD-7): Anxiety symptoms were quantified using the GAD-7, a seven-item screening tool designed to identify probable cases of generalized anxiety disorder and assess symptom severity over the preceding two weeks [22]. Participants responded to items (e.g., “Feeling nervous, anxious or on edge”) on a 4-point Likert scale ranging from 0 (not at all) to 3 (nearly every day). Total scores range from 0 to 21. For clinical interpretation, scores were categorized as follows: 0–4 (minimal anxiety), 5–9 (mild anxiety), 10–14 (moderate anxiety), and 15–21 (severe anxiety). In this study, consistent with established clinical practice, a cut-off score of ≥10 was used to identify clinically relevant anxiety symptoms [22].

Patient Health Questionnaire-9 (PHQ-9): Depressive symptoms were evaluated using the PHQ-9, which consists of nine items corresponding to the diagnostic criteria for major depressive disorder [23]. Participants rated the frequency of symptoms (e.g., “Little interest or pleasure in doing things”) over the past two weeks on a scale from 0 (not at all) to 3 (nearly every day). The total score ranges from 0 to 27. Severity was categorized based on established thresholds: 0–4 (minimal or no depression), 5–9 (mild depression), 10–14 (moderate depression), 15–19 (moderately severe depression), and 20–27 (severe depression). A score of ≥10 was considered the threshold for clinically significant depressive symptoms [23].

Participants completed the questionnaires anonymously during routine outpatient visits at the HIV/AIDS Centre or via secure electronic communication. Responses were collected and processed using Qualtrics software (Qualtrics, Provo, UT, USA) in a fully anonymized format to ensure privacy and reduce social desirability biases.

### 2.3. Study Variables

To ensure methodological transparency, data were categorized into three primary domains:

Sociodemographic Variables: These included age (years), sex (male or female), nationality (Slovak or Ukrainian), and displacement status (specifically for Ukrainian participants).

Clinical and HIV-Specific Variables: Time since HIV diagnosis: The duration (in years) from the first laboratory-confirmed HIV diagnosis to the point of study enrollment. Mode of HIV transmission: Participants were categorized into three primary groups based on the reported route of infection: men who have sex with men (MSM), heterosexual contact, and people who inject drugs (PWID). Clinical Staging: HIV infection stage was classified according to the Centers for Disease Control and Prevention (CDC) clinical categories (Stage A, B, or C) [9]. CART Status: Defined as current adherence to a cART. Virological Status: Plasma HIV-1 RNA viral load (VL) was used as the primary marker of treatment efficacy.

Mental Health Outcomes: Total scores and severity categories (minimal: 0–4, mild: 5–9, and clinically significant: ≥10) derived from the GAD-7 (anxiety) and PHQ-9 (depression) instruments, as detailed in Section 2.2.

All data were extracted from standardized electronic medical records maintained at the Centre, ensuring consistency and accuracy across both study cohorts.

### 2.4. Statistical Analysis

Continuous variables were expressed as means ± standard deviations (SD) or medians with interquartile ranges (IQR), depending on the distribution of the data. Categorical variables were expressed as absolute numbers and percentages. Differences between categorical variables were analyzed using the chi-square test or Fisher’s exact test when expected cell counts were small. Continuous variables were compared using one-way analysis of variance (ANOVA) where appropriate. When expected cell counts were <5, Fisher’s exact test was used. For mental health outcomes, GAD-7 and PHQ-9 scores were categorized into three severity groups according to Section 2.2. and differences between groups were evaluated using chi-square tests. Categorization was used to facilitate clinical interpretation of symptom severity. All statistical tests were two-sided and statistical significance was defined as *p* < 0.05. Statistical analyses were performed using IBM SPSS Statistics for Windows, version 20.0 (IBM Corp., Armonk, NY, USA). Due to the sample size distribution, multivariable modeling was not performed to avoid model overfitting; bivariate comparisons were prioritized.

### 2.5. AI Statement

During the preparation of this manuscript, the authors used the Gemini 3 Flash (Google, Google, Mountain View, CA, USA) and ChatGPT 5.2 (OpenAI, San Francisco, CA, USA) generative AI tool for the purposes of spelling correction, grammatical refinement, and language polishing of the original draft. After using this tool, the authors reviewed and edited the content as needed and take full responsibility for the final content of the published article.

## 3. Results

### 3.1. Cohort Characteristics

In this cross-sectional study, 137 people living with HIV were included, comprising 69 participants from Ukraine and 68 from Slovakia (Table 1). The Ukrainian cohort had a balanced sex distribution (35 men [50.72%] and 34 women [49.28%]), whereas the Slovak cohort was predominantly male (59 men [86.76%] and 9 women [13.24%]). This difference in sex distribution between cohorts was statistically significant (χ^2^ = 19.02, *p* < 0.001) (Table 1). The low number of Slovak female participants reflects the overall proportion of women living with HIV followed at our center, which is less than 10% and mirrors demographics of PLHIV in Slovakia [9]. The mean age of participants was comparable between cohorts. Age ranged from 39.5 ± 10.5 years in Ukrainian men to 44.2 ± 7.0 years in Ukrainian women (Table 1). When age was compared across the four sex- and country-specific subgroups (Slovakia—men, Slovakia—women, Ukraine—men, Ukraine—women), no statistically significant difference was observed (ANOVA *p* = 0.2438) (Table 2). Additional sex-stratified sociodemographic characteristics are summarized in Table 3.

### 3.2. Mode of Transmission and Clinical Staging

Modes of HIV transmission differed markedly between cohorts (χ^2^ = 71.22, *p* < 0.001) (Table 1 and Table 3). Among Ukrainian participants, heterosexual transmission predominated (53 cases; 76.81%), followed by PWID (8 cases; 11.59%), men who have sex with men (MSM) transmission (6 cases; 8.70%), and vertical transmission (2 cases; 2.90%). In contrast, the Slovak cohort was characterized primarily by MSM transmission (51 cases; 75.00%), followed by heterosexual transmission (14 cases; 20.59%) and undetermined transmission routes (3 cases; 4.41%) (Table 1). Sex-stratified analysis demonstrated that MSM transmission occurred exclusively among men and was particularly frequent among Slovak men (86.44%), while heterosexual transmission predominated among women in both cohorts (Ukraine—women 82.35%; Slovakia—women 100%) (Table 3). Clinical stage distribution also differed between cohorts, although the difference did not reach statistical significance Clinical stage distribution also differed between cohorts, although the difference did not reach statistical significance (χ^2^ = 7.10, *p* = 0.069) (Table 1). CDC stage A was more frequent in Slovakia (64.71%) than in Ukraine (42.03%), whereas CDC stage C occurred more commonly among Ukrainian patients (49.28%) compared with Slovak patients (29.41%) (Table 1).

### 3.3. Treatment Outcomes

All participants in both cohorts were receiving cART (Table 1). Viral suppression rates were high in both groups. VL levels below 200 copies/mL were observed in 63 Ukrainian participants (91.30%) and 64 Slovak participants (94.12%), with no statistically significant difference between cohorts (*p* = 0.761) (Table 1). Using a stricter virological threshold, VL below 40 copies/mL were present in 55 Ukrainian patients (79.71%) and 58 Slovak patients (85.29%) (Table 1). Sex-stratified analysis demonstrated similarly high suppression rates across subgroups, with VL < 200 copies/mL observed in 88.57% of Ukrainian men, 94.12% of Ukrainian women, 93.22% of Slovak men, and 100% of Slovak women (Table 3). Coinfection with hepatitis C virus (HCV) was significantly more common among Ukrainian participants than among Slovak (34.78% vs. 2.94%; χ^2^ = 20.56, *p* < 0.001) (Table 1). Drug abuse reported in the medical history did not differ significantly between cohorts (21.74% vs. 11.76%; *p* = 0.182) (Table 1). Similarly, the prevalence of previously treated depression or anxiety did not differ significantly between groups (Table 1).

### 3.4. Mental Health Comparisons

Severity of anxiety symptoms assessed using the GAD-7 scale did not differ significantly between the Ukrainian and Slovak cohorts (χ^2^ = 1.761, *p* = 0.4145) (Table 4). Minimal anxiety symptoms (score 0–4) were observed in 54.41% of Slovak participants and 43.48% of Ukrainian participants. Mild symptoms (score 5–9) occurred in 26.47% and 30.43% of participants, respectively, while moderate-to-severe anxiety symptoms (score ≥ 10) were present in 19.12% of Slovak and 26.09% of Ukrainian participants (Table 4). Depressive symptom severity measured using the PHQ-9 scale also did not differ significantly between cohorts (χ^2^ = 0.533, *p* = 0.7661) (Table 5). Sex-stratified analyses showed no statistically significant differences in anxiety severity between men and women within either cohort (Ukraine *p* = 0.346; Slovakia *p* = 0.345) (Table 6). In contrast, depressive symptom severity differed between sexes within the Ukrainian cohort, where higher PHQ-9 severity categories were more frequently observed among women than men (*p* = 0.0478) (Table 7). No statistically significant sex difference in depressive severity was observed within the Slovak cohort (*p* = 0.220) (Table 7). When anxiety symptoms were dichotomized using the threshold GAD-7 ≥ 10, clinically relevant anxiety symptoms were present in 19.12% of Slovak participants and 26.09% of Ukrainian participants, without a statistically significant difference between cohorts (χ^2^ = 0.74, *p* = 0.39).

## 4. Discussion

This study provides a comprehensive evaluation of the clinical and psychosocial status of war-displaced Ukrainian PLHIV compared to a host-country cohort in Slovakia. Our findings demonstrate that despite the profound challenges of forced migration and ongoing conflict, the Slovak healthcare system has successfully maintained a high standard of care, achieving virtually 100% cART coverage and high viral suppression rate among displaced patients. While the overall prevalence of anxiety and depressive symptoms did not differ significantly between the Ukrainian and Slovak cohorts, a critical gender-specific disparity emerged within the displaced group, with Ukrainian women exhibiting significantly higher levels of depressive symptoms compared to men. These results underscore the importance of integrating gender-sensitive mental health support into the standard clinical management of displaced PLHIV to ensure long-term treatment adherence and wellbeing.

### 4.1. Gendered Stress Pathways in Conflict Settings

Women displaced by war frequently assume primary caregiving responsibilities, experience separation from partners, and face economic instability [11,14]. These chronic stressors are strongly linked to depressive pathways characterized by role overload and diminished future orientation. Research in conflict settings indicates that daily stressors may mediate mental health outcomes more strongly than direct trauma exposure [20,21]. The observed depressive disparity among Ukrainian women aligns with literature demonstrating higher depression prevalence among women living with HIV [2,3,18]. The intersection of gender, chronic illness, and displacement likely magnifies baseline vulnerability.

### 4.2. Biomedical Stability Versus Psychosocial Divergence

Our finding of high viral suppression rates among war-displaced Ukrainian PLHIV is a testament to the effectiveness of the ‘immediate linkage to care’ model. This high rate of treatment efficacy aligns with recent data from other European host countries, such as Poland and Germany, where rapid integration into national healthcare systems yielded similar virological outcomes [7,11]. However, our results stand in contrast to historical data on forced migration from conflict zones, where treatment interruptions often led to significantly lower suppression rates due to fragmented care [16,25]. The success observed in the Slovak cohort can be attributed to the elimination of financial barriers through mandatory public health insurance and the centralized nature of care in Bratislava, which minimizes the ‘lost-to-follow-up’ phenomenon often seen in decentralized systems. Furthermore, the high baseline cART coverage among Ukrainians upon arrival suggests a resilient pre-war HIV program in Ukraine, which provided a solid foundation for continued care in exile.”

### 4.3. Clinical Stage C and Structural Stress

The higher proportion of CDC stage C among Ukrainian patients may reflect wartime interruption of care and delayed linkage to services [26], particularly given that the majority of these patients had already been previously diagnosed and confirmed in Ukraine. This interpretation is also consistent with epidemiological data indicating that late HIV diagnosis remains common in Ukraine [9,27]. Beyond biomedical severity, a history of advanced disease may reinforce perceived fragility and uncertainty, particularly among women with caregiving responsibilities. However, this interpretation should be considered hypothesis-generating. Because trauma exposure, duration of displacement, and prior treatment interruption were not systematically quantified, causal inferences regarding the relationship between advanced clinical stage and depressive burden cannot be established. Future longitudinal analyses incorporating clinical history and psychosocial variables would be necessary to clarify these pathways.

### 4.4. Structural Vulnerability and Intersectionality

The disparate mental health outcomes observed in our study must be interpreted through the lens of structural vulnerability—the risk that an individual experiences due to their position within a given social and political hierarchy. For Ukrainian PLHIV in Slovakia, this vulnerability is inherently intersectional, as it arises from the overlapping impacts of forced displacement, the chronic nature of HIV infection, and gender-based social expectations.

Our findings highlight that while the Slovak healthcare system provides a robust “structural safety net” by offering universal access to cART through mandatory health insurance [7,9], this biological protection does not fully mitigate the social and structural stressors of exile. As noted by Kashnitsky et al. [13], refugees often navigate a “legal and administrative labyrinth” that can exacerbate psychological distress. In our cohort, this was particularly evident among women, who frequently experience a “compounded burden” of intersectionality. They are not only managing a stigmatized chronic illness but are also navigating the precarity of displaced status while fulfilling traditional caregiving roles for children and elderly relatives, often in the absence of male family members who remained in Ukraine [8]. While the Slovak cohort’s predominantly single status reflects the specific social profile of host-country MSM, the marital status of Ukrainian participants (52.2% married) introduces a distinct, gendered stressor. For these women, being ‘married’ often translates into a status of enforced separation due to wartime mobilization in Ukraine. Furthermore, the concept of structural vulnerability explains why clinical stability may coexist with significant psychological morbidity. The “medicalization” of the refugee response—focusing primarily on viral load suppression and drug dispensing—may overlook the structural determinants of health, such as housing stability, language barriers, and the loss of social capital. The identified gender gap in anxiety and depression suggests that the current care model, while virologically effective, may be “gender-blind” to the specific structural pressures faced by women [11,12,15]. To move beyond limited interpretation, it is essential to recognize that intersectional vulnerabilities require intersectional solutions. Addressing the mental health of displaced PLHIV in Slovakia necessitates a transition from purely clinical management to a more holistic, supportive care model that integrates psychological screening and social support systems tailored to the unique structural position of displaced women.

Our findings provide empirical evidence of a syndemic interaction within the displaced Ukrainian cohort. While the Slovak healthcare system successfully addresses the biomedical component of the HIV epidemic, as evidenced by high viral suppression rates, the psychosocial component remains significantly elevated, particularly among women. This suggests that the syndemic cycle is not fully broken by clinical stabilization alone. The intersection of gender and displacement acts as a catalyst within this syndemic, where the ‘daily stressors’ of exile interact with the chronic burden of HIV to produce the observed higher severity of depressive symptoms. Addressing these outcomes requires a syndemic-oriented approach that integrates mental health services as a core component of routine HIV care. Our findings—showing a gap between high viral suppression and elevated depressive symptoms in women —reflect the sociological status of the ‘outsider within’ [15,16]. While Ukrainian PLHIV are clinically ‘insiders’ with 100% cART coverage, they remain ‘outsiders’ within the host country’s social fabric, navigating language barriers and lost social capital. This ‘outsider within’ position is most acute for women, who are biometrically stabilized but psychosocially isolated by the compounded burden of displacement and caregiving roles [16].

### 4.5. Clinical Implications and Strategies to Reduce Gender Disparities

The findings of this study have direct implications for clinical practice in humanitarian and displacement settings. First, systematic depression screening using validated instruments such as the PHQ-9 should be routinely implemented within HIV care for displaced populations, particularly among women. Depression is consistently associated with poorer cART adherence and reduced virological suppression, and its treatment has been shown to improve HIV-related outcomes [2,3,4,5]. Screening should not be episodic but longitudinal, recognizing that depressive symptoms may emerge or intensify over time as acute survival concerns transition into chronic socioeconomic stress.

Second, HIV clinics serving displaced populations should integrate low-threshold psychosocial support pathways. These may include on-site psychological counselling, structured referral networks with community mental health services, and culturally sensitive group-based or peer-supported interventions. Evidence from conflict-affected settings indicates that community-oriented and psychosocial interventions can mitigate internalizing symptoms and reduce the impact of daily stressors that mediate mental health outcomes [20,21].

Third, gender-sensitive care models are warranted. For displaced women living with HIV, caregiving burden, economic insecurity, and family separation represent key stress mediators [11,14]. Clinical teams should incorporate brief assessments of caregiving strain, housing stability, and social support during routine HIV visits. Where possible, flexible appointment scheduling, childcare facilitation, and linkage to social services may mitigate structural barriers that disproportionately affect women.

Beyond the clinical setting, mitigation of gender disparities requires coordinated social-level responses. The legal status of persons living with HIV in the Slovak Republic is safeguarded by a general prohibition of discrimination on the grounds of “other status” or disability, which constitutes the fundamental framework for protection against unequal treatment. At the same time, the Criminal Code regulates criminal liability for intentional transmission, for which it provides a term of imprisonment ranging from three to ten years, as well as for negligent transmission, where the term of imprisonment ranges from one to five years, depending on the nature and seriousness of the unlawful conduct [28]. Within the legal order of Ukraine, HIV is explicitly recognised as a specific ground of discrimination, and the legal framework is further complemented by a separate statute governing the prevention of and response to HIV [29]. Policies in Slovakia ensuring stable legal status, access to employment, language integration programs, and childcare support are likely to have downstream mental health benefits. Structural vulnerability frameworks emphasize that political and socioeconomic arrangements fundamentally shape health outcomes, particularly in contexts of displacement and conflict [12]. Intersectoral collaboration between healthcare providers, social services, non-governmental organizations, and public health authorities is therefore essential to address structural stressors that cannot be resolved within the medical system alone. Importantly, framing depressive vulnerability among displaced women solely as an individual psychological issue risks obscuring the structural determinants underlying the disparity. Intersectionality theory highlights how overlapping identities—including gender, migration status, and HIV status—produce compounded vulnerability that requires multidimensional responses [16]. Interventions should therefore combine individual-level mental health support with structural-level stabilization strategies.

Our findings highlight a high standard of clinical care, with virtually 100% of participants in both cohorts receiving cART. This universal coverage likely contributed to the high rates of viral suppression observed in our study, underscoring the effectiveness of the Slovak healthcare system’s response to the influx of displaced persons.

### 4.6. Epidemiological Profiles, Comorbidities, and Therapy

However, notable differences were observed in the epidemiological and clinical profiles of the two groups. The Ukrainian cohort presented with a significantly higher proportion of heterosexually transmitted infections and a higher representation of women, contrasting with the predominantly MSM (men who have sex with men) profile of the Slovak group. Furthermore, a larger percentage of Ukrainian patients were diagnosed at a more advanced clinical stage. These divergences in transmission routes and clinical presentation reflect the distinct national HIV epidemics and should be considered when evaluating the psychosocial challenges faced by each group. The more advanced clinical stages in the displaced cohort may also indicate barriers to early diagnosis or disruptions in healthcare continuity prior to their arrival in the host country. The significantly higher prevalence of HCV coinfection among Ukrainian participants further underscores the complexity of their clinical management. This finding aligns with the historically concentrated nature of the HIV epidemic in Ukraine and suggests that displaced PLHIV may require integrated care models that address both HIV and viral hepatitis, alongside mental health screening [6,7].

### 4.7. Study Contribution and Novelty

This study contributes novel evidence from a real-world European humanitarian context by directly comparing war-displaced and host-country PLHIV within a shared healthcare system. Unlike prior research that has focused either on refugee mental health or HIV outcomes separately, our analysis integrates epidemiological staging, virological stability, and gender-differentiated mental health patterns within a unified framework. The finding that overall mental health severity did not differ between displaced and host-country patients—despite conflict exposure—while sex-specific depressive vulnerability emerged within the displaced cohort, provides a more nuanced understanding of resilience and risk in humanitarian HIV care. By situating clinical staging, gender, and displacement within structural vulnerability and intersectionality frameworks, this study moves beyond descriptive epidemiology toward an explanatory model linking biomedical stability with psychosocial differentiation. These insights may inform the design of integrated HIV and mental health services in other European settings receiving displaced populations.

### 4.8. Limitations

This study has several limitations. The cross-sectional design precludes causal inference and limits temporal interpretation of associations between displacement, clinical staging, and depressive symptoms. Approximately 50% of eligible participants completed the mental health survey, introducing potential selection bias.

Furthermore, this study has limitations regarding the demographic composition and size of the control group. The small sample size of Slovak female participants limited our ability to perform a robust comparative gender-based analysis within the host-country cohort. However, this distribution reflects the real-world epidemiological situation in the Slovak Republic, where the vast majority of patients followed at our center are MSM. This mirrors the long-term national HIV trends, where the MSM transmission category remains predominant, in contrast to the more heterogenous transmission profile seen in the Ukrainian cohort [9,27]. While these divergence in risk groups and gender representation are inherent to the respective national epidemics, they should be taken into account when interpreting the comparative mental health outcomes. Given the inclusion of the entire eligible clinical population via consecutive sampling, the study achieved the maximum possible power within the constraints of the national epidemiological landscape.

Multivariable modelling was not performed due to the relatively small sample size, particularly within the Slovak female subgroup, which would result in unstable estimates and potential model overfitting. Because reliable multivariable regression typically requires a substantially larger number of outcome events per predictor variable, the present analysis was restricted to descriptive and bivariate comparisons.

The single-centre design may limit generalizability to other European healthcare settings. Future studies with larger multi-centre cohorts would allow more robust multivariable analyses to better disentangle the independent contributions of gender, displacement status, and clinical characteristics.

To mitigate these constraints in future research, several strategies should be considered. To improve response rates and reduce potential selection bias, upcoming studies could implement targeted participation incentives or utilize peer-led recruitment strategies, which might better engage hard-to-reach or stigmatized populations. Furthermore, expanding to a multi-center longitudinal design would not only increase the statistical power for gender-based comparisons but also allow for a more robust assessment of how mental health trajectories evolve as displacement shifts from an acute to a chronic stressor.

## 5. Conclusions

This study demonstrates that displaced PLHIV can achieve and maintain high rates of viral suppression and cART coverage within a structured host-country healthcare system, even during an active conflict and displacement crisis. Despite the significant clinical challenges, such as a higher prevalence of advanced CDC stages and HCV coinfection among the Ukrainian cohort, the biomedical outcomes were comparable to those of the Slovak host population. However, our findings highlight a critical divergence in mental health outcomes. While overall anxiety and depression scores did not differ significantly between countries, sex-stratified analysis revealed a specific vulnerability among Ukrainian women, who exhibited significantly higher depressive symptom severity compared to Ukrainian men. This disparity, not observed in the Slovak cohort, suggests that the intersection of displacement, war-related trauma, and gender-specific social roles creates a unique psychological burden. In conclusion, clinical management of displaced PLHIV must extend beyond a purely biomedical focus. It is imperative to integrate routine, longitudinal, and gender-sensitive mental health screening into standard HIV care. Addressing the specific psychosocial needs of displaced women is essential to ensure long-term health equity and the continued success of HIV treatment in refugee populations.

## Figures and Tables

**Table 1:** Sociodemographic and clinical characteristics of people living with HIV in Ukraine and Slovakia (cross-sectional study). Values are *n* (%) unless stated otherwise.

Variable and Category	Ukraine *n* = 69 (%)	Slovakia *n* = 68 (%)	*p*
Men	35 (50.72%)	59 (86.76%)	*p* < 0.001
Women	34 (49.28%)	9 (13.24%)	
Mode of transmission			
heterosexual	53 (76.81%)	14 (20.59%)	*p* < 0.001
MSM	6 (8.70%)	51 (75.00%)	
PWID	8 (11.59%)	0 (0.00%)	
vertical	2 (2.90%)	0 (0.00%)	
blood transfusion	0 (0.00%)	0 (0.00%)	
undetermined	0 (0.00%)	3 (4.41%)	
Stage of HIV infection/CDC			
stage A	29 (42.03%)	44 (64.71%)	0.069
stage B	3 (4.35%)	2 (2.94%)	
stage C	34 (49.28%)	20 (29.41%)	
undetermined	3 (4.35%)	2 (2.94%)	
Marital status			
single	15 (21.74%)	55 (80.88%)	*p* < 0.001
married	36 (52.17%)	4 (5.88%)	
divorced	11 (15.94%)	3 (4.41%)	
widowed	2 (2.90%)	0 (0.00%)	
undetermined	5 (7.25%)	6 (8.82%)	
Comorbidities			
HCV coinfection	24 (34.78%)	2 (2.94%)	<0.001
Drug abuse in anamnesis	15 (21.74%)	8 (11.76%)	0.182
Therapy depression/anxiety in anamnesis	3 (4.35%)	7 (10.29%)	0.313
Treatment and virology			
VL < 200 copies/mL	63 (91.30%)	64 (94.12%)	0.761
VL < 40 copies/mL	55 (79.71%)	58 (85.29%)	
Receiving cART treatment	69 (100.00%)	68 (100.00%)	

cART, combined antiretroviral therapy; CDC, Centers for Disease Control and Prevention; HCV, hepatitis C virus; MSM, men who have sex with men; *n*, number of participants; PWID, people who inject drugs; *p*, *p*-value; VL, viral load.

**Table 2:** Age distribution of people living with HIV by country and sex.

Group	*n*	Mean ± SD (Years)	Median (IQR) (Years)
Slovakia—men	59	41.9 ± 10.3	40.0 (36.0–47.0)
Slovakia—women	9	42.4 ± 7.7	40.0 (38.0–46.0)
Ukraine—men	35	39.5 ± 10.5	41.0 (34.0–46.5)
Ukraine—women	34	44.2 ± 7.0	44.0 (42.0–48.8)

ANOVA *p*-value: 0.2438—individual groups did not differ statistically significantly; SD, standard deviation; IQR, interquartile range.

**Table 3:** Sex-stratified sociodemographic and clinical characteristics of people living with HIV in the Slovak and Ukrainian cohorts.

Variable and Category	Ukraine—Men (*n* = 35)	Ukraine—Women (*n* = 34)	Slovakia—Men (*n* = 59)	Slovakia—Women (*n* = 9)	*p*
Mode of transmission					
heterosexual	25 (71.43%)	28 (82.35%)	5 (8.47%)	9 (100.00%)	*p* < 0.001
MSM	6 (17.14%)	0 (0.00%)	51 (86.44%)	0 (0.00%)	
PWID	3 (8.57%)	5 (14.71%)	0 (0.00%)	0 (0.00%)	
vertical	1 (2.86%)	1 (2.94%)	0 (0.00%)	0 (0.00%)	
blood transfusion	0 (0.00%)	0 (0.00%)	0 (0.00%)	0 (0.00%)	
undetermined	0 (0.00%)	0 (0.00%)	3 (5.08%)	0 (0.00%)	
Stage of HIV infection/CDC					
stage A	15 (42.86%)	14 (41.18%)	36 (61.02%)	8 (88.89%)	*p* = 0.18
stage B	2 (5.71%)	1 (2.94%)	2 (3.39%)	0 (0.00%)	
stage C	17 (48.57%)	17 (50.00%)	19 (32.20%)	1 (11.11%)	
stage undetermined	1 (2.86%)	2 (5.88%)	2 (3.39%)	0 (0.00%)	
Marital status					
single	12 (34.29%)	3 (8.82%)	51 (86.44%)	4 (44.44%)	*p* < 0.001
married	17 (48.57%)	19 (55.88%)	3 (5.08%)	1 (11.11%)	
divorced	2 (5.71%)	9 (26.47%)	2 (3.39%)	1 (11.11%)	
widowed	1 (2.86%)	1 (2.94%)	0 (0.00%)	0 (0.00%)	
undetermined	3 (8.57%)	2 (5.88%)	3 (5.08%)	3 (33.33%)	
Comorbidities					
HCV coinfection	13 (37.14%)	11 (32.35%)	2 (3.39%)	0 (0.00%)	*p* < 0.001
Drug abuse	7 (20.00%)	8 (23.53%)	8 (13.55%)	0 (0.00%)	0.42
Depression/anxiety therapy	1 (2.86%)	2 (5.88%)	4 (6.78%)	3 (33.33%)	0.12
Treatment and virology					
VL < 200 copies/mL	31 (88.57%)	32 (94.12%)	55 (93.22%)	9 (100.00%)	0.78
VL < 40 copies/mL	28 (80.00%)	27 (79.41%)	49 (83.05%)	9 (100.00%)	0.55
Receiving cART	35 (100.00%)	34 (100.00%)	59 (100.00%)	9 (100.00%)	

cART, combined antiretroviral therapy; CDC, Centers for Disease Control and Prevention; HCV, hepatitis C virus; MSM, men who have sex with men; *n*, number of participants; PWID, people who inject drugs; *p*, *p*-value; VL, viral load.

**Table 4:** Anxiety severity (GAD-7) among people living with HIV in Ukraine and Slovakia.

Severity (GAD-7)	Slovakia *n* (%) (*n* = 68)	Ukraine *n* (%) (*n* = 69)
0–4 (minimal)	37 (54.41%)	30 (43.48%)
5–9 (mild)	18 (26.47%)	21 (30.43%)
≥10 (moderate–severe)	13 (19.12%)	18 (26.09%)
Statistical Test	Chi-square = 1.761; *p* = 0.4145	

GAD-7, Generalized Anxiety Disorder-7 scale; *n*, number of participants; *p*, *p*-value.

**Table 5:** Depressive symptom severity (PHQ-9) among people living with HIV in Ukraine and Slovakia.

Severity (PHQ-9)	Slovakia *n* (%) (*n* = 68)	Ukraine *n* (%) (*n* = 69)
0–4 (minimal)	27 (39.71%)	24 (34.78%)
5–9 (mild)	20 (29.41%)	24 (34.78%)
≥10 (moderate–severe)	21 (30.88%)	21 (30.43%)
Statistical Test	Chi-square = 0.533; *p* = 0.7661	

PHQ-9, Patient Health Questionnaire-9 scale; *n*, number of participants; *p*, *p*-value.

**Table 6:** Anxiety severity (GAD-7) by sex in Ukrainian and Slovak cohorts.

Severity	UA Men (*n* = 35)	UA Women (*n* = 34)	SK Men (*n* = 59)	SK Women (*n* = 9)
0–4 (minimal)	18 (51.43%)	12 (35.29%)	34 (57.63%)	3 (33.33%)
5–9 (mild)	10 (28.57%)	11 (32.35%)	15 (25.42%)	3 (33.33%)
≥10 (moderate–severe)	7 (20.00%)	11 (32.35%)	10 (16.95%)	3 (33.33%)
*p*-value	UA: *p* = 0.346		SK: *p* = 0.345	

GAD-7, Generalized Anxiety Disorder-7 scale; UA, Ukraine; SK, Slovakia; *n*, number of participants; *p*, *p*-value.

**Table 7:** Depressive symptom severity (PHQ-9) by sex in Ukrainian and Slovak cohorts.

Severity	UA Men (*n* = 35)	UA Women (*n* = 34)	SK Men (*n* = 59)	SK Women (*n* = 9)
0–4 (minimal)	17 (48.57%)	7 (20.59%)	25 (42.37%)	2 (22.22%)
5–9 (mild)	9 (25.71%)	15 (44.12%)	18 (30.51%)	2 (22.22%)
≥10 (moderate–severe)	9 (25.71%)	12 (35.29%)	16 (27.12%)	5 (55.56%)
*p*-value	UA: *p* = 0.0478		SK: *p* = 0.220	

PHQ-9, Patient Health Questionnaire-9 scale; UA, Ukraine; SK, Slovakia; *n*, number of participants; *p*, *p*-value.

## Data Availability

The data presented in this study are available on reasonable request from the corresponding author. The data are not publicly available due to privacy and ethical restrictions, as they contain sensitive information regarding the clinical status of people living with HIV.

## Abbreviations

The following abbreviations are used in this manuscript:

AIDS	Acquired immunodeficiency syndrome
ANOVA	Analysis of variance
cART	Combined antiretroviral therapy
CDC	Centers for Disease Control and Prevention
GAD-7	Generalized Anxiety Disorder-7 scale
HCV	Hepatitis C virus
HIV	Human immunodeficiency virus
IQR	Interquartile range
MSM	Men who have sex with men
*n*	Number of participants/Sample size
*p*	*p*-value (probability)
PHQ-9	Patient Health Questionnaire-9
PLHIV	People living with HIV
PWID	People who inject drugs
RNA	Ribonucleic acid
SD	Standard deviation
SK	Slovakia
UA	Ukraine
VL	Viral load
